# Chromogranin A is a reliable serum diagnostic biomarker for pancreatic neuroendocrine tumors but not for insulinomas

**DOI:** 10.1186/1472-6823-14-64

**Published:** 2014-08-07

**Authors:** Xin-Wei Qiao, Ling Qiu, Yuan-Jia Chen, Chang-Ting Meng, Zhao Sun, Chun-Mei Bai, Da-Chun Zhao, Tai-Ping Zhang, Yu-Pei Zhao, Yu-Li Song, Yu-Hong Wang, Jie Chen, Chong-Mei Lu

**Affiliations:** 1 Department of Gastroenterology, Peking Union Medical College Hospital, Peking Union Medical College, Chinese Academy of Medical Sciences, Beijing, 100730, People’s Republic of China; 2 Department of Clinical Laboratory, Peking Union Medical College Hospital, Peking Union Medical College, Chinese Academy of Medical Sciences, Beijing, 100730, People’s Republic of China; 3 Key Laboratory of Endocrinology (Ministry of Health), Department of Endocrinology, Peking Union Medical College Hospital, Peking Union Medical College, Chinese Academy of Medical Sciences, Beijing 100730, People’s Republic of China; 4 Department of Oncology, Peking Union Medical College Hospital, Peking Union Medical College, Chinese Academy of Medical Sciences, Beijing 100730, People’s Republic of China; 5 Department of Pathology, Peking Union Medical College Hospital, Peking Union Medical College, Chinese Academy of Medical Sciences, Beijing 100730, People’s Republic of China; 6 Department of Surgery, Peking Union Medical College Hospital, Peking Union Medical College, Chinese Academy of Medical Sciences, Beijing 100730, People’s Republic of China; 7 Department of Gastroenterology, The First Affiliated Hospital of Sun Yat-sen University, Guangzhou 510000, People’s Republic of China

## Abstract

**Background:**

Pancreatic neuroendocrine tumors (PNETs) are a group of rare tumors. Chromogranin A (CgA) was considered as the most practical and useful serum tumor marker in PNET patients. But peripheral blood levels of CgA are not routinely tested in Chinese patients with PNETs. This study was to assess the diagnostic value of CgA in Chinese patients with PNETs especially in patients with insulinomas.

**Methods:**

Eighty-nine patients with PNETs including 57 insulinomas and 32 non-insulinoma PNETs as well as 86 healthy participants were enrolled in this study between September 2003 and June 2013. Serum levels of CgA were measured by ELISA method. Expression of CgA protein was detected in 26 PNET tissues including 14 insulinomas by immunohistochemical staining.

**Results:**

Serum levels of CgA in 89 PNET patients were significantly higher than that in healthy controls (*P* = 7.2 × 10^−9^). Serum levels of CgA in 57 patients with insulinomas (median 64.8 ng/ml, range 25–164) were slightly higher than the levels in healthy controls (median 53.4 ng/ml, range 39–94) but much lower than the levels in 32 patients with non-insulinoma PNETs (median 193 ng/ml, range 27–9021), *P* = 0.001. The serum CgA levels were reduced in 16 of 17 patients with insulinomas after tumor resection. ROC curve showed that CgA values at 60 ng/ml distinguished patients with insulinomas from healthy controls but its sensitivity and specificity were 66.7% and 73.3%, respectively. In contrast, CgA values at 74 ng/ml distinguished patients with non-insulinoma PNETs from healthy controls, and the sensitivity and specificity were 65.6% and 91.9%, respectively. Except for two insulinomas with negative staining of CgA, 12 insulinoma tissues showed positive staining of CgA.

**Conclusion:**

CgA is a reliable serum diagnostic biomarker for PNETs but not for insulinomas.

## Background

Pancreatic neuroendocrine tumors (PNETs) are a group of rare tumors. The prevalence and incidence have increased over the past 3 decades [1-5]. The clinical presentations of PNETs are very complicated due to excess of gut peptides produced by functioning PNETs while symptoms of nonfunctioning PNETs (NF) are obscure [1,3]. In addition, most of PNETs could be biologically aggressive [1,3,6]. Thus, the earlier and accurate diagnosis of PNET is important to facilitate surgical resection and/or to initiate appropriate medical management such as molecular targeted therapy, biotherapy and other intensive care.

Chromogranin A (CgA) is a 46-kDa glycoprotein, member of the granin family, exists within all type of neurons, normal neuroendocrine cells and is expressed in NET cells [7,8]. Over the past 2 decades, many studies reported and confirmed that CgA was a reliable diagnostic biomarker for NETs including gastroentero-pancreatic NETs (GEP-NET) [1,3,5-20] and also might be a prognostic biomarker for NETs [21]. Moreover, several studies showed that peripheral blood levels of CgA were increased in endocrine-associated tumors, for example, breast cancer [22] and prostate cancer [23,24]. Recently, elevated serum/plasma levels of CgA were found in a number of non-endocrine solid tumors, such as hepatic carcinomas [25,26] and pancreatic cancer [27]. Examination of CgA levels could be used not only for diagnosis but also for prognostic evaluation in these tumors [21,23,25,27].

In recent ENETS and NANETS consensus guidelines, CgA was considered as the most practical and useful serum tumor marker in PNET patients [28,29]. Some studies suggested that testing blood CgA should be mandatory for NET diagnosis [7]. However, few attention has been paid to the insulinoma which is the most common type of functioning PNETs [7,8,14,15,17,19,21,30-32]. In addition, peripheral blood levels of CgA are not routinely tested in Chinese patients with GEP-NET. Using CgA for clinical diagnosis has not been officially approved by Sino Food Drug Administration (SFDA) because little data have been reported.

Thus, the present study is to verify the utility of CgA in diagnosis of PNETs, focusing on its diagnostic value in insulinoma. We found that serum levels of CgA were not significantly elevated in patients with insulinomas, compared to the higher levels of CgA in other PNETs. This finding was rarely reported in previous studies.

## Methods

### Ethics statement

This study was approved by the Scientific Ethics Committee of Peking Union Medical College Hospital and the First Affiliated Hospital of Sun Yat-sen University. Participants provided their written informed consent to participate in this study. The Scientific Ethics Committee of both hospitals approved the consent procedure.

### Patients and samples collection

Eighty-nine Chinese patients with PNETs including 57 insulinomas (one with extensive hepatic metastases), and 32 non-insulinoma PNETs (8 gastrinomas, 4 glucagonomas, 1 VIPoma and 19 NF) as well as 86 healthy participants were enrolled in this study at Peking Union Medical College Hospital and the First Affiliated Hospital of Sun Yat-sen University, between September 2003 and June 2013. The diagnostic criteria for PNETs were reported previously [33-39]. Briefly, the tumors were mainly localized by computed tomography with contrast, magnetic resonance imaging, endoscopic ultrasound and somatostatin receptor scintigraphy. All patients did not suffer with inflammatory diseases (such as inflammatory bowel disease, chronic atrophic gastritis), and the renal, hepatic or cardiac insufficiency was excluded. The patients did not take proton pump inhibitors (PPIs) or histamine 2 receptor blockers as well as somatostatin analogues. The pathological diagnosis was made by 2 experienced pathologists. We analyzed tumor grade in 54 tumors according to ENETS-WHO guideline [40] and analyzed stage in 84 patients according to the ENETS guideline [40].

Before surgery or treatment, blood samples were obtained in 89 fasted patients with PNETs. In 17 patients with insulinomas, blood samples were postoperatively collected during 3rd to 7th days after resection. The blood samples from 86 healthy participants (median age 43 years, 38 male) were collected after overnight fasting. Serum were isolated and stored in −80°C.

### Detecting of serum levels of CgA

Serum levels of CgA of patients with PNETs and healthy controls were measured by ELISA method with a commercial kit (Chromoa assay; CIS Bio International, France), according to the manufacturer’s protocol. Most of samples were duplicated tested, and some of samples were checked in another experiment. Ten samples were double examined in two different labs (one in Beijing and another lab in Guangzhou).

### Detecting of CgA expression in tumor tissues

Expression of CgA protein was detected in 26 sections of paraffin-embedded PNETs tissues, including 14 insulinomas, 2 gastrinomas and 10 NF as well as their paired pancreatic (duodenal) tissues by immunohistochemical staining (IHC) with anti-CgA (AC-0037, clone EP38, Epitomics, Inc, Burlingame, CA) at a 1:100 dilution. The criteria of semi-quantitative grading of IHC was similar to our previous report [36,39], i.e. (−) means no positive staining in tumor cells; (±) < 20% tumor cells shown positive staining, (+) ≥20% but < 50% tumor cells shown positive staining; (++) ≥50% but < 75% tumor cells shown positive staining; (+++) ≥ 75% tumor cells shown positive staining. We defined < 20% tumor cells with staining of CgA as negative staining, i.e. (−) and (±).

### Statistical analysis

To verify the diagnostic value of serum CgA, receiver operating characteristic (ROC) curves were plotted, and the area under the curve (AUC) was calculated. SPSS statistics software version 13.0 was used for statistical analysis. Mann–Whitney method was used to compare the CgA levels between each group of patients and healthy controls. Fisher exact test or Chi’s test were used to analyze our data. Two-tailed test was used in all of statistic analysis. *P* < 0.05 was considered statistically significant.

## Results

### Clinicopathological characteristics

We studied 89 PNET patients, 73 patients underwent curative surgery and 16 patients did not undergo operation. All tumors were well differentiated. The clinicopathological characteristics of 89 patients with PNETs and 57 patients with insulinomas were summarized in Table 1.

**Table 1:** Clinicopathological characteristics of PNET patients

**Clinicopathological features of patients with PNETs**		**Number**
Gender n = 89 (%)	Male	36 (40.4)
	Female	53 (59.6)
	Male : Female	1:1.47
Age (years) at diagnosis, n = 87	Median (range)	47 (16–74)
PNET subtype n = 89 (%)	Insulinoma	57 (64.0)
	Non-insulinoma	32 (36.0)
	NF	19 (21.3)
	gastrinoma	8 (9.0)
	glucagonoma	4 (4.5)
	VIPoma	1 (1.1)
Inherited or sporadic PNETs, n = 89 (%)	Sporadic	86 (96.6)
	MEN-1 associated	3 (3.4)
Surgery or not, n = 89 (%)	Resection	73 (82.0)
	Unresection	16 (18.0)
Primary tumor location, n = 77 (%)	Pancreatic head/neck	40 (51.9)
	Pancreatic body/tail	36 (46.8)
	duodenum	1 (1.3)
Tumor size (cm) median (range) n = 82		1.6 (0.8-8)
	Insulinoma n = 56	1.5 (0.8-4)
	Non-insulinoma n = 26	4.3 (1.5-8)
Metastasis or not, n = 83 (%)	No	65 (78.3)
	Yes	18 (21.7)
Grade, n = 54 (%)	G1	37 (68.5)
	G2	15 (27.8)
	G3	2 (3.7)
Stage, n = 84 (%)	I	39 (46.4)
	II	22 (26.2)
	III	5 (6.0)
	IV	18 (21.4)
**Clinicopathological features of patients with insulinomas**		**Number**
Gender n = 57 (%)	Male	25 (43.9)
	Female	32 (56.1)
	Male : Female	1:1.28
Age (years) at diagnosis, n = 57	Median (range)	47 (16–74)
Inherited or sporadic PNETs, n = 57 (%)	Sporadic	55 (96.5)
	MEN-1 associated	2 (3.5)
Surgery or not, n = 57 (%)	Resection	54 (94.7)
	Unresection	3 (5.3)
Primary tumor location, n = 56 (%)	Pancreatic head/neck	30 (53.6)
	Pancreatic body/tail	26 (46.4)
Tumor size (cm) median (range) n = 56		1.5 (0.8-4)
Metastasis or not, n = 57 (%)	No	56 (98.2)
	Yes	1 (1.8)
Grade, n = 35 (%)	G1	31 (88.6)
	G2	4 (11.4)

### CgA Serum Levels in PNET Patients and ROC curves

**Figure 1:**
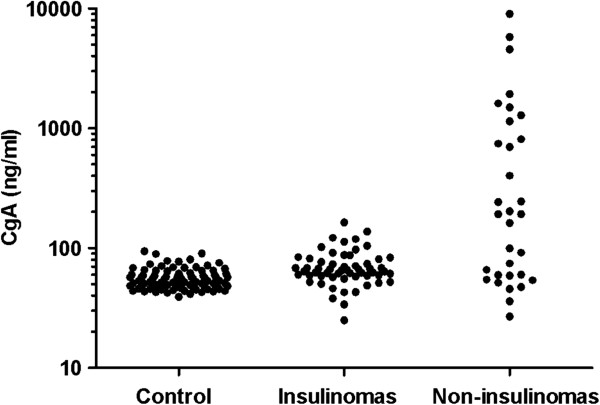
**Serum concentrations of CgA in patients with insulinomas and non-insulinoma PNETs and in healthy controls.** Individual levels were presented as dots. The results were plotted logarithmically to accommodate extreme values. The serum CgA levels were slightly elevated in 57 patients with insulinomas while the CgA levels were significantly increased in 32 patients with non-insulinoma PNETs. The serum levels of CgA in patients with insulinomas (median 64.8 ng/ml) were significantly lower than that in the patients with non-insulinoma PNETs (median 192.5 ng/ml), *P* = 0.001.

The median values of CgA levels in 86 healthy controls, 57 patients with insulinomas and 32 patients with non-insulinoma PNETs were 53.4 ng/ml (range 39.1 – 94.1 ng/ml), 64.8 ng/ml (range 25.0 – 164.2 ng/ml) and 192.5 ng/ml (range 26.9 – 9020.7 ng/ml), respectively (Figure 1). Serum levels of CgA in 89 PNET patients were significantly higher than that in healthy controls (*P* = 7.2 × 10^−9^). Compared with the serum levels of CgA in the healthy controls, the CgA levels were significantly elevated in 32 patients with non-insulinoma PNETs (*P* = 3.7 × 10^−7^). In contrast, the levels of CgA in 57 patients with insulinomas were just slightly higher than that in healthy participants (median 64.8 ng/ml vs. 53.4 ng/ml), see Figure 1. The serum levels of CgA in patients with insulinomas (median 64.8 ng/ml) were significantly lower than that in the patients with non-insulinoma PNETs (median 192.5 ng/ml), *P* = 0.001, Figure 1.

**Figure 2:**
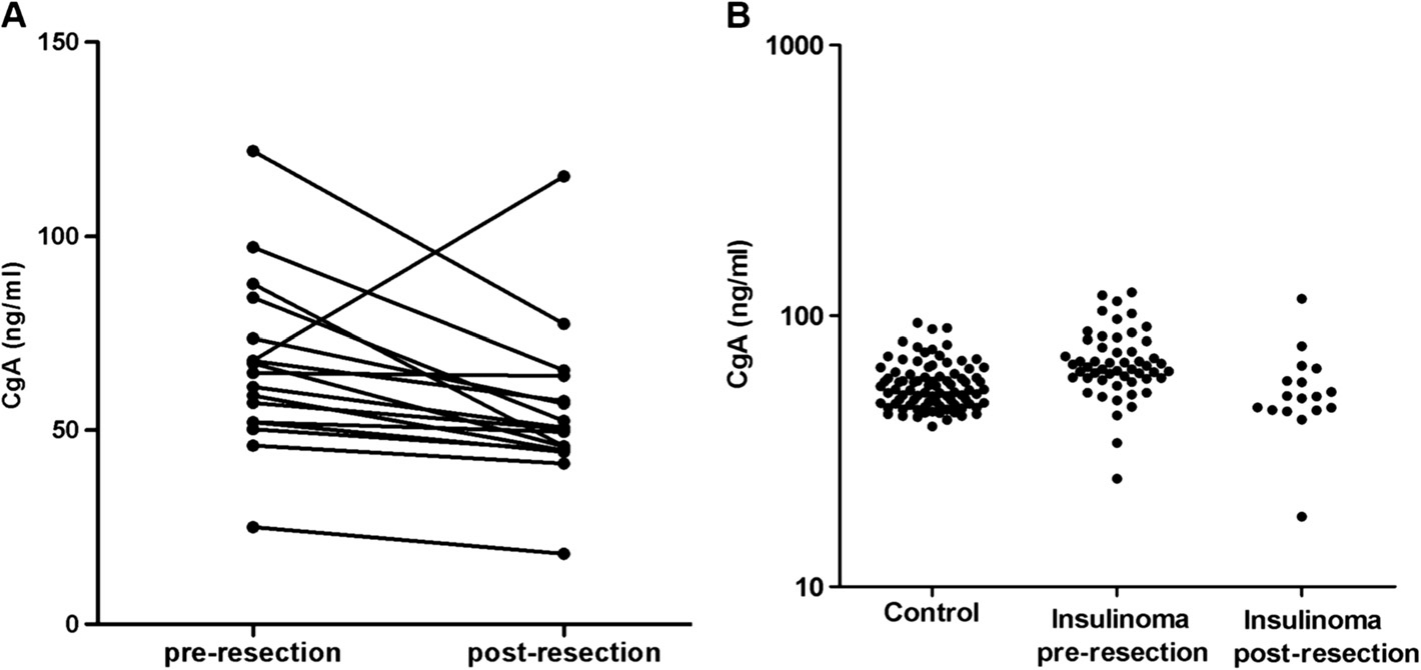
**Decreased CgA levels in patients with insulinomas after tumor resection.** Figure 2**A** The serum levels of CgA were significantly reduced in 16 of 17 patients with insulinomas after resection, median 64.8 ng/ml vs. 50.4 ng/ml, *P* = 0.003; Figure 2**B** The serum levels of CgA in healthy controls and pre-/post-operative serum levels of CgA in patients with insulinomas. Individual levels are presented as dots.

**Figure 3:**
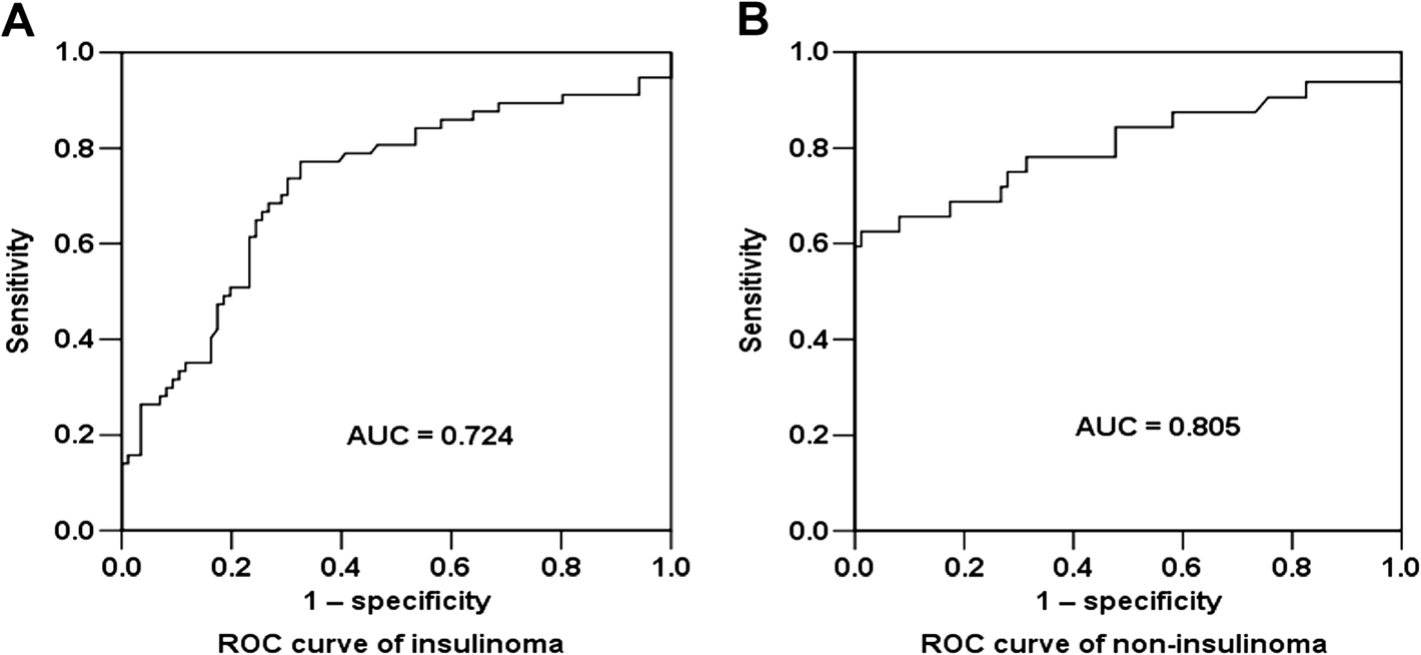
**Diagnostic accuracy of CgA in PNETs.** Figure 3**A** ROC curves of CgA levels in patients with insulinomas (n = 57) and in patients with non-insulinoma PNETs (Figure 3**B**, n = 32) versus healthy participants (n = 86). AUC: the area under the curve.

Although the levels of CgA in patients with insulinomas were not elevated significantly, it was interesting that the serum levels of CgA were decreased in 16 of 17 patients with insulinomas after tumor resection (Figure 2A, median 64.8 ng/ml vs. 50.4 ng/ml, *P* = 0.003). The postoperative levels of CgA in patients with insulinomas were almost the same as the levels in healthy controls (Figure 2B, median 50.4 ng/ml vs. 53.4 ng/ml).ROC curve showed that CgA values at 60.4 ng/ml distinguished patients with insulinomas from healthy controls with the sensitivity of 66.7% and specificity was 73.3%, AUC was 0.724 (Figure 3A). In contrast, CgA values at 73.9 ng/ml distinguished patients with non-insulinoma PNETs from healthy controls, with a sensitivity and specificity were 65.6% and 91.9%, respectively, AUC was 0.805 (Figure 3B). These findings suggested that the serum levels of CgA in patients with insulinomas were not obviously elevated and CgA was not a reliable diagnostic biomarker for insulinomas due to the low specificity.

### Correlation of CgA levels with clinicopathological characteristics in patients with PNETs and insulinomas

**Figure 4:**
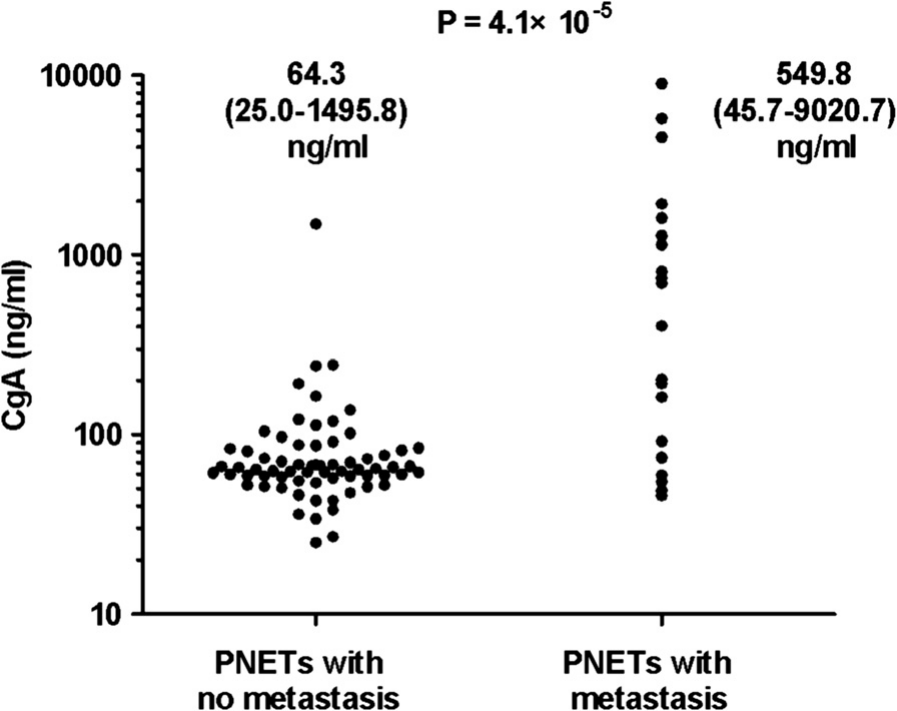
**Comparing CgA levels in patients with tumor metastasis with the levels in patients with localized tumors.** Individual levels were presented as dots. The serum CgA levels in 18 patients with tumor metastasis were significantly higher than that in 65 patients with localized tumors, *P* = 4.1 × 10^−5^.

**Figure 5:**
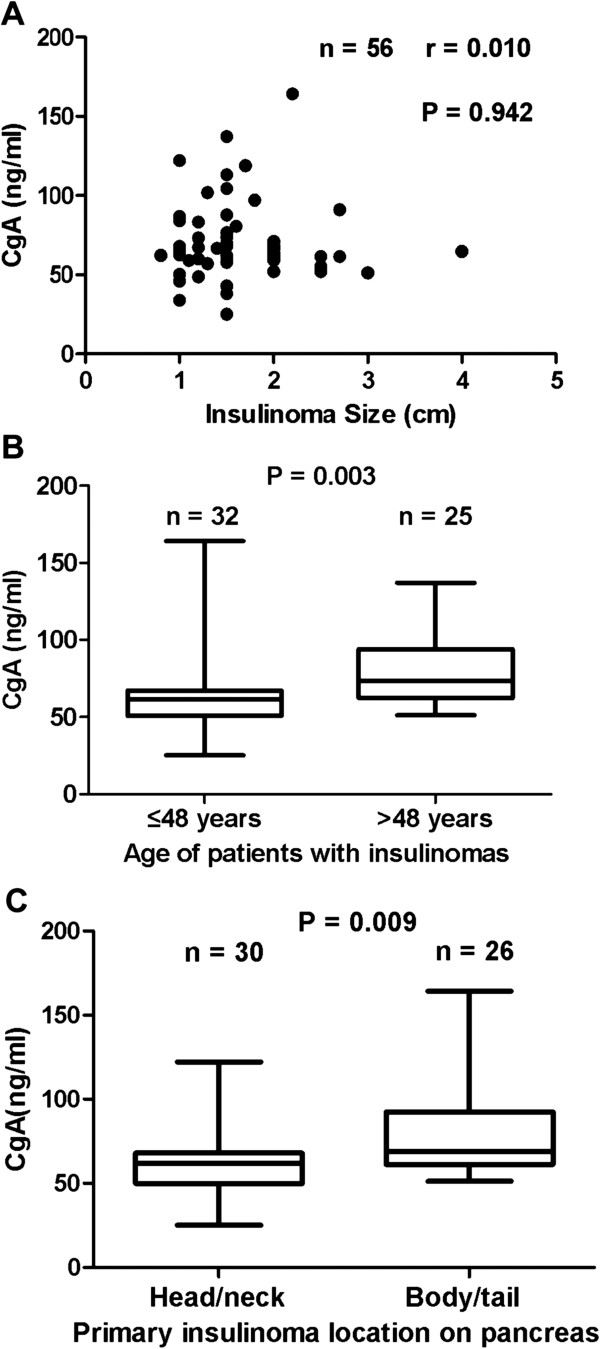
**Correlation of serum CgA levels with insulinoma size, primary location and age of patients with insulinomas.** Figure 5**A** The serum levels of CgA did not correlate with insulinoma size (n = 56). Individual CgA levels are presented as dots. Figure 5**B** The serum levels of CgA in elder patients with insulinomas were significantly higher than that in younger patients (n = 57). The ends of the error bars represent the minimum and maximum of CgA levels in different groups. Figure 5**C** The serum levels of CgA were significantly associated with primary location of insulinomas (*P* = 0.009).

We correlated the CgA levels with clinicopathological features in patients with PNETs as well as insulinomas, respectively (Table 2). We found that the serum CgA levels in 18 patients with tumor metastases were significantly higher than that in 65 patients with localized tumors (median value, 549.8 ng/ml vs. 64.3 ng/ml, respectively, *P* = 4.1 × 10^−5^), see Figure 4. The serum levels of CgA in PNETs patients were not significantly associated with gender, tumor size and grade but associated with age, primary tumor location and stage, see Table 2. Furthermore, we correlated the CgA levels with clinicopathological features in patients with insulinomas. The serum levels of CgA in patients with insulinomas were not significantly associated with gender, tumor size (Figure 5A), grade and stage, but significantly associated with age (Figure 5B) and primary tumor location (Figure 5C), see Table 2.

**Table 2:** Correlation of CgA levels with clinicopathological features in patients with PNETs and insulinomas

**Clinicopathological features of patients with PNETs**		**CgA levels (ng/ml) median (range)**	**P value**
Gender	Male, n = 36	67.9 (46.1-9020.7)	0.394
	Female, n = 53	67.2 (25.0-4572.3)	
Age (years)	≤48, n = 49	64.8 (25.0-5772.5)	0.039
	>48, n = 38	81.2 (26.9-9020.7)	
Primary tumor location on pancreas	Head/neck, n = 41	62.5 (25.0-5772.5)	0.019
	Body/tail, n = 36	81.9 (45.7-9020.7)	
Tumor size (cm)	n = 82, 0.8 - 8,	67.0 (25.0-9020.7)	0.545
	r = 0.068,		
Grade	G1, n = 37	67.3 (33.9-811.9)	0.103
	G2, n = 15	74.3 (26.9-1931.1)	
	G3, n = 2	323.7 (244.4-403.0)	
Stage	I, n = 39	67.2 (25.0-137.2)	0.003
	II, n = 22	61.6 (51.2-241.7)	
	III, n = 5	244.4 (26.9-811.9)	
	IV, n = 18	302.8 (45.7-9020.7)	
**Clinicopathological features of patients with insulinomas**		**CgA levels (ng/ml) median (range)**	**P value**
Gender	Male, n = 25	66.8 (46.1-164.2)	0.072
	Female, n = 32	62.6 (25.0-122.0)	
Age (years)	≤48, n = 32	61.4 (25.0-164.2)	0.003
	>48, n = 25	73.3 (51.2-137.2)	
Primary tumor location on pancreas	Head/neck	61.9 (25.0-122.0)	0.009
	n = 30		
	Body/tail	68.9 (51.2-164.2)	
	n = 26		
Tumor size (cm)	n = 56, 0.8 - 4,	64.3 (25.0-164.2)	0.942
	r = 0.01		
Grade	G1, n = 31	64.8 (33.9-164.2)	0.795
	G2, n = 4	62.5 (57.9-104.4)	
Stage	Stage I, n = 39	67.2 (25.0-137.2)	0.215
	Stage II, n = 15	61.6 (51.2-164.2)	

### The CgA levels in patients with localized insulinomas and in patients with localized non-insulinomas

**Figure 6:**
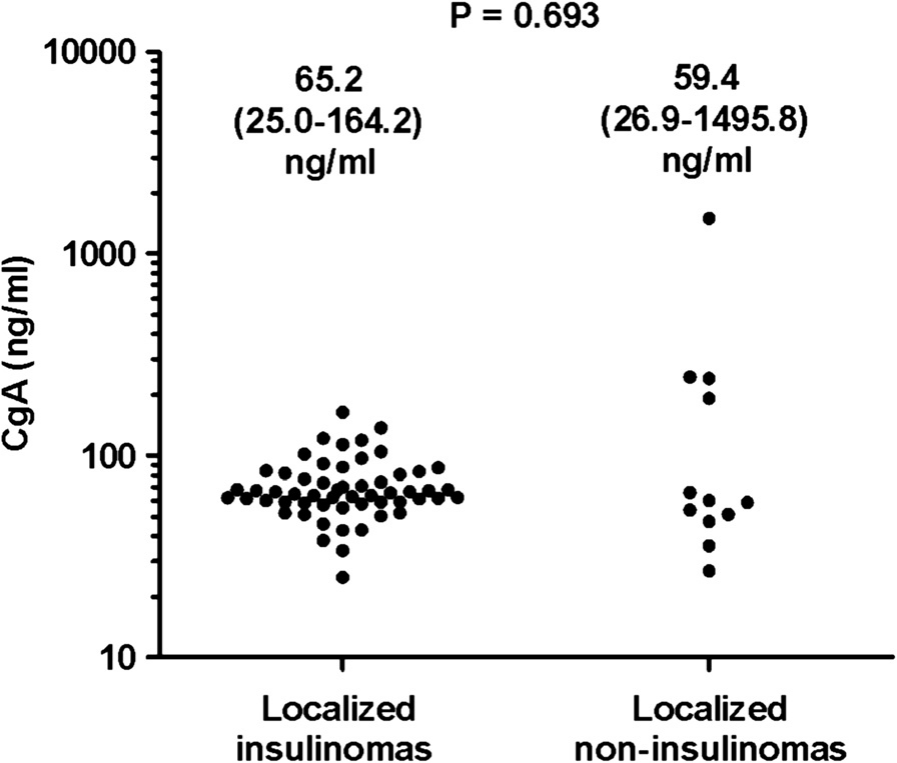
**Comparing CgA levels in benign insulinomas with the levels in patients with localized non-insulinomas.** The serum levels of CgA in 56 patinets with localized insulinomas were similar to the levels in 12 patients with localized non-insulinomas, *P* = 0.693. It can be seen that 3 of 56 patients with localized insulinomas had the CgA levels more than 120.8 ng/ml (i.e. 2 fold more than cut-off values for insulinoma), whereas 4 of 12 patients with localized non-insulinomas had CgA levels more than 242 ng/ml (2 fold more than cut-off values for non-insulinoma).

Our data above showed that CgA levels in patients with tumor metastases were significantly higher than that in patients with localized tumors and only 1 patient with metastasic insulinoma was included in our study. We compared serum CgA levels in 56 localized insulinoma with the CgA levels in 12 patients with localized non-insulinomas. The serum levels of CgA in both groups of patients were similar, 65.2 ng/ml vs. 59.4 ng/ml, *P* = 0.693 (Figure 6). As we mentioned above, the ROC curve showed that CgA cut-off values was 60.4 ng/ml for insulinomas and 73.9 ng/ml for non-insulinomas, respectively. We noticed that only 3 of 56 patients with localized insulinomas had the CgA levels more than 120.8 ng/ml (i.e. two fold more than cut-off values for insulinoma), whereas 4 of 12 patients with localized non-insulinomas had CgA levels more than 242 ng/ml (also two fold more than cut-off values for non-insulinoma), *P* = 0.015 (Fisher exact), see Figure 6, indicating the patients with localized non-insulinomas had a more frequency of remarkable elevation of CgA than that in the patients with localized insulinomas.

### The expression of CgA in PNETs tissues

**Figure 7:**
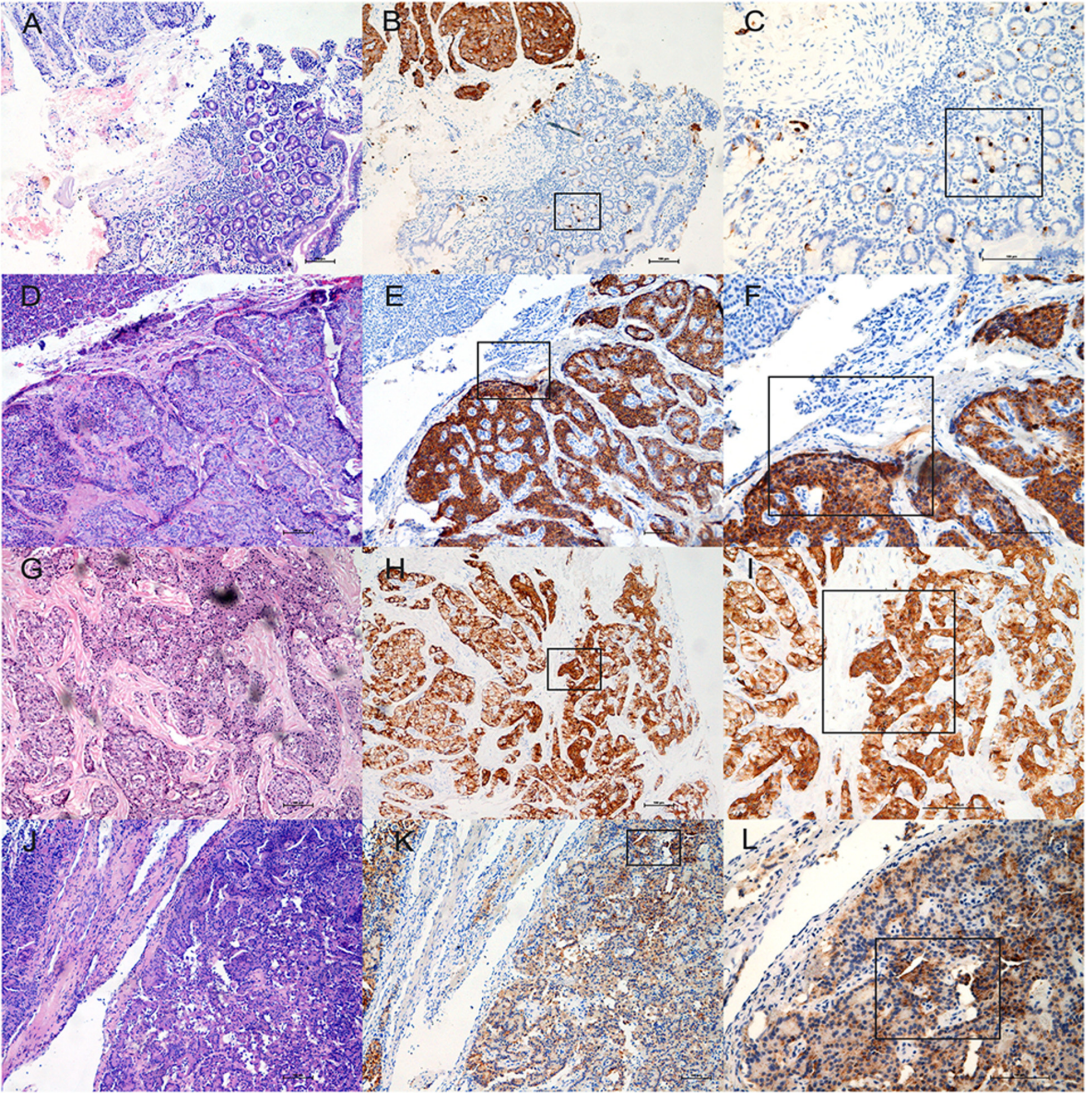
**Representative examples of CgA expression in PNETs and their paired tissues.** Left panel: HE staining; middle panel: CgA IHC, 10×; right panel: CgA IHC, 20×. Figure 7**A** shown HE staining of a gastrinoma located in duodenum, Figure 7**B** shown the strong expression of CgA protein in gastrinoma tissue and Figure 7**C** shown scattered CgA positive cells in non-tumoral duodenum tissue. Figure 7**D**, Figure 7**G** and Figure 7**J** shown HE staining of 3 different insulinomas, respectively. Figure 7**E-F**, Figure 7**H-I** and Figure 7**K-L** shown the very strong signals of CgA, strong signals of CgA and medium signals of CgA in 3 different insulinomas, respectively. In contrast, no expression of CgA can be seen in the interstitial tissues within the tumor and in the pancreatic exocrine tissues.

Because the serum CgA levels were not elevated in patients with insulinomas, we wanted to determine whether the protein of CgA was expressed in tumor tissues or not. We observed strong or medium expression of CgA in 12 of 14 insulinomas as well as in 11 of 12 non-insulinoma PNETs, as detected by IHC (Figure 7). Two of 14 insulinomas and 1 of 12 non-insulinoma PNETs had very weak IHC staining of CgA protein. The expression of CgA in PNET tissues was not associated with serum levels of CgA in corresponding patients. These data suggested that most of insulinoma cells could synthesize a great amount of CgA protein.

## Discussion

Circulating CgA levels have been confirmed to be useful diagnostic marker for NETs, with a high specificity and sensitivity [1,3,5-19,28]. In the present study, we verified the diagnostic value of serum CgA in a series of patients with non-insulinoma PNETs, in agreement with previous studies. The sensitivity and specificity were 65.6% and 91.9%, respectively, similar to the rate of 67% and 96% when using CIS Bio kits, as Ardill and Erikkson described [12]. Similar to previous studies [9-11,14,16,41,42], we found that CgA levels in patients with gastrinomas were much higher than those in patients without gastrin-secreting PNETs.

The interesting finding in the study was that serum CgA levels were not elevated in patients with insulinomas, including one patient with extensive liver metastases (48 ng/ml). Most of previous studies on PNETs did not clarify this unusual biochemical feature of insulinoma, the most common subtype of PNETs [7,8,13-16,19,20,31] but Wouter de Herder pointed out in a review that blood levels of CgA were rarely slightly elevated in subjects with insulinomas [43] and Portela-Gomes GM et al. mentioned in a review that well-differentiated NETs expressed CgA epitopes except insulinomas [44]. A recently published guideline which was revised by the UK and Ireland Neuroendocrine Tumor Society and the British Society of Gastroenterology addressed that CgA would not raised in benign insulinomas [45]. Moreover, Nobels et al. studied more than 200 NETs and found that serum CgA levels were rarely slightly elevated in patients with insulinomas (elevated in 2 of 21 patients, range 63–236 ng/ml, upper cut-off value was 220 ng/ml) [9]. Another study showed that CgA levels were not elevated in 5 cases of insulinomas [13]. In present study, we focused on insulinomas and our findings were very similar to their data which showed only a small part of patients with insulinomas (7/57, 12%) had a slightly increased level of CgA (>100 ng/ml, the highest level: 164 ng/ml). With a relatively low specificity (73%), serum CgA was not a reliable and practicable biomarker for diagnosis of insulinoma. This finding is important. Some studies suggested that testing CgA levels should be mandatory for PNETs diagnosis [7]. Furthermore, according to recent North American Neuroendocrine Tumor Society (NANETS) and European Neuroendocrine Tumor Society (ENETS) consensus guidelines [28,29] as well as ESMO guidelines for NETs diagnosis [5], CgA was considered as a general biomarker for NETs, and CgA can be used as a marker in patients with both Functional PNET and NF-PNET [28,29]. Insulinoma is the most common subtype of functioning PNETs [31,35,37,46]. However, whether serum levels of CgA should be tested in patients with insulinomas has not been well clarified in those guidelines for NETs diagnosis. Our data and previous reports [9,13] showed that insulinoma could be an exception for measuring serum levels of CgA for diagnostic purpose. It maybe not necessary to test CgA levels in patients with insulinomas although this issue needs to be further validated in more cases and in multiple clinical centers. In addition, using different commercial kits or assay could be useful to further validate our findings because the antibodies used in different assay were raised against the different domain or epitopes of the CgA molecular [12,15,17,47].

The underlying mechanism of low CgA levels in patients with insulinomas is not clear. Nobels et al. speculated the serum levels of CgA were only slightly elevated in subjects with small NETs, such as insulinomas, pituitary adenomas [9]. However, there is disagreement in the literatures whether the serum CgA levels correlate with the extent of NETs or size of these tumors [7,21,32,48]. In present study, we did not observe the correlation between the tumor size and CgA levels in PNETs (*P* = 0.545) and in insulinomas alone (*P* = 0.942). Some of non-insulinoma PNETs with relatively small size still had very high serum levels of CgA, for example, CgA level was 4572 ng/ml in a gastrinoma of 1.5 cm in size, and the highest CgA level in present study was more than 9000 ng/ml in a glucagonoma of 2.5 cm in size. Furthermore, one patient with a NF of 3.5 cm in size had a CgA level of 5772 ng/ml whereas another patient with a NF of 8 cm in size had a CgA level of 59 ng/ml.

Many previous studies [10,13,15,41,42,49] and our present data showed that CgA levels in patients with NETs metastases were much higher than that in patients with localized NETs. Thus, it may hypothesized that few metastases in insulinomas would be the reason for low serum level of CgA in insulinomas (only one metastatic insulinoma in our serial). We found CgA levels in localized insulinomas were similar to that in localized non-insulinomas, *P* = 0.693. This might imply the above hypothesis could be partly true. In fact, most of insulinomas (>90%) are benign, absent metastasis in majority of insulinomas is one of the main characteristics of this unique tumor, and in our hospital, more than 95% of insulinomas are benign [37]. However, we noticed that the rate of elevated CgA levels in patients with localized non-insulinomas was significantly higher than that in patients with localized insulinomas, *P* = 0.015. In addition, one report showed that in 9 of the 10 patients with gastrinoma, CgA values were raised, even in the absence of metastasis [41]. These data suggested that metastasis could be one of determinant factors for high levels of CgA in PNETs, but not the only one. The tumor subtype could be another important determinant for CgA serum levels. Nevertheless, more patients with metastatic insulinomas were needed to validate the low levels of CgA in insulinomas although it might be quite difficult to do so due to the limited numbers of malignant insulinomas.

It was reported that pancreastatin, a CgA-derived peptide (CgA residues 250–301) with biological activity, inhibited the releasing of insulin by islet beta cells [7,50] and insulinoma cell line [51]. Gayen et al. [52] observed an inverse relationship between pancreastatin and insulin. This CgA-derived peptide might antagonize the effect of insulin via the Akt/FOXO-1 and, administration of insulin could result in low plasma levels of pancreastatin in mice (the basal pancreastatin level dropped significantly following insulin injection). In majority of insulinomas, a great deal amount of insulin is secreted by tumor cells. We speculate that high levels of insulin in patients with insulinomas might inhibit the secretion of CgA in these tumor cells. A recent study demonstrated that insulin and proinsulin were released in patients with insulinomas in response to arterial calcium stimulation, whereas CgA was not released [53].

One research suggested that CgA targeted to secretory granules in association with protein secretogranin III, a member of granin family, in pituitary and pancreatic endocrine cells [54]. If other proteins such as secretogranin III were broken down, the secretion of CgA would be disrupted. The mechanisms of hormones and peptides secretion were very complicated, it maybe concerned with molecular cellular biology and the alterations of tumor cell functions.

Other than the low blood levels of CgA in insulinomas, the biomedical behaviors of insulinomas were quite different from other PNETs. For example, its low rate of malignancy (<10%), the relatively low rate of positive Octreotide scintigraphy can be identified in benign insulinomas comparing with non-insulinoma PNETs because many insulinomas do not express somatostatin receptor subtypes [35]. All of these unusual features of insulinomas indicated that unique molecular cellular aspects and/or functions existed in insulinoma cells.

In this study, we have observed that most of insulinoma tissues (12/14) were shown strong positive staining for CgA, indicating that insulinoma cells were able to synthesize the CgA protein. This aspect of insulinoma was similar to non-insulinoma PNETs. However, only small part of the protein might be secreted into blood by the insulinoma cells because the CgA levels were not elevated and, the CgA levels were significantly reduced in 16 patients after tumor resection (from median 64.8 ng/ml to median 50.4 ng/ml, *P* = 0.003).

It is hard to explain why the serum level of CgA was postoperatively elevated in one patient who had normal liver and kidney functions. This patient did not suffer with other disease or take PPI or H2 receptor blocker. The sample was detected repeatedly and the same results were obtained.

In conclusion, our findings suggested that CgA is not a reliable biomarker for insulinomas, hence, examination of blood CgA levels could not be recommended in patients with insulinoms according to Nobels’ [9] and our data. The mechanisms underlying low serum levels of CgA in insulinomas would appear to warrant further investigation.

## Conclusion

The study revealed that the circulating CgA levels in patients with insulinomas were not obviously elevated, although we did validate the diagnostic value of serum CgA in a series of patients with non-insulinoma PNETs.

## Competing interests

The authors declare that they have no conflict of interest.

## Authors’ contributions

YJC designed the study. XWQ, LQ, YJC, CTM, ZS, CMB, TPZ, YPZ, YHW and JC collected the samples and detected serum levels of CgA. XWQ, YJC, DCZ, YLS carried out the immunohistochemical staining. XWQ, LQ, CML and YJC analyzed the data and performed the statistical analysis. XWQ and YJC drafted the manuscript. All authors read and approved the final manuscript.

## Pre-publication history

The pre-publication history for this paper can be accessed here:

http://www.biomedcentral.com/1472-6823/14/64/prepub
